# Optimizing strategies for meningococcal C disease vaccination in Valencia (Spain)

**DOI:** 10.1186/1471-2334-14-280

**Published:** 2014-05-21

**Authors:** Lina Pérez-Breva, Rafael J Villanueva, Javier Villanueva-Oller, Luis Acedo, Francisco Santonja, José A Moraño, Raquel Abad, Julio A Vázquez, Javier Díez-Domingo

**Affiliations:** 1 Centro Superior de Investigación en Salud Pública-Fundación para el Fomento de la Investigación Sanitaria y Biomédica de Comunidad Valencia (CSISP-FISABIO), Avd. Cataluña, Valencia, Spain; 2 Instituto de Matemática Multidisciplinar, Edificio 8G 2° Floor, Universitat Politècnica de Valéncia, Camino de Vera s/n, 46022 Valencia, Spain; 3 Centro de Estudios Superiores Felipe II, Aranjuez, Madrid, Spain; 4 Departamento de Estadística e Investigación Operativa, Universidad de Valencia, Valencia, Spain; 5 Instituto de Salud Carlos III, 28029 Majadahonda, Madrid, Spain

**Keywords:** Meningococcal C conjugate vaccines, Seroprotection study, Agent-based modelling, Vaccination programs

## Abstract

**Background:**

Meningococcal C (MenC) conjugate vaccines have controlled invasive diseases associated with this serogroup in countries where they are included in National Immunization Programs and also in an extensive catch-up program involving subjects up to 20 years of age. Catch-up was important, not only because it prevented disease in adolescents and young adults at risk, but also because it decreased transmission of the bacteria, since it was in this age group where the organism was circulating. Our objective is to develop a new vaccination schedule to achieve maximum seroprotection in these groups.

**Methods:**

A recent study has provided detailed age-structured information on the seroprotection levels against MenC in Valencia (Spain), where vaccination is routinely scheduled at 2 months and 6 months, with a booster dose at 18 months of age. A complementary catch-up campaign was also carried out in n for children from 12 months to 19 years of age. Statistical analyses of these data have provided an accurate picture on the evolution of seroprotection in the last few years.

**Results:**

An agent-based model has been developed to study the future evolution of the seroprotection histogram. We have shown that the optimum strategy for achieving high protection levels in all infants, toddlers and adolescents is a change to a 2 months, 12 months and 12 years of age vaccination pattern. If the new schedule were implemented in January 2014, high-risk subjects between 15-19 years of age would have very low seroprotection for the next 6 years, thereby threatening the program.

**Conclusions:**

High protection levels and a low incidence of meningococcal C disease can be achieved in the future by means of a cost-free change in vaccination program. However, we recommend a new catch-up program simultaneous to the change in regular vaccination program.

## Background

Neisseria meningitidis serogroup C (MenC) was the leading cause of meningococcal disease in Spain in the late 1990s. Invasive disease was most frequent in infants and toddlers, and also peaked in adolescents and young adults. Carriage studies showed it to be this older age group in which MenC was carried [1,2].

In the year 2000, when a conjugated MenC vaccine became available, the latter was included in the Spanish National Immunization Program with a three-dose schedule at 2, 4, and 6 months, and a catch-up regimen in all the population up to 19 years of age. The objective of the catch-up was not only to afford direct protection of the adolescents but also to decrease carriage and therefore secure indirect protection of the population. After the beginning of the vaccination campaigns, the incidence of meningococcal disease decreased by 87*%* in Spain [3].

In 2005, a seroprotection study showed the short-term protection afforded by primovaccination in infants [4], and therefore a booster dose was added in the second year of life.

There are no accurate data on the coverage of the catch-up campaign in Spain, which proved different in each of the 17 Autonomous Communities – thereby rendering the epidemiology different in different areas of the country [5]. In Valencia, catch-up coverage was age dependent, ranging from 67*%* in the oldest subjects to 87*%* for subjects under 5 years of age [6]. In 2011, a seroprevalence study was carried out in Valencia in all age groups from three years of age. This included 1880 blood samples in which serum bactericidal assay (SBA) was carried out using rabbit complement (SBAr) [7]. A mathematical analysis of these results allowed a reliable protective rating in Spain, taking into account the differences among subjects included in the primovaccinated or booster groups and in the catch-up policy.

The protection conferred by the MenC vaccines is not long lasting, and in infants and toddlers the immunity disappears relatively fast [8]. This waning protection can be studied by the dynamics of the serum bactericidal assays (SBA). Seroepidemiological studies performed 3 to 6 years after a single dose of MenC vaccine proved that the percentage of seroprotected individuals (SBA ≥ 1:8) decreased, and that the loss of protection was faster in infants than in adolescents. Recently, the United Kingdom and Spain have considered giving a further booster dose to adolescents in order to keep the young adults protected when the subjects that were vaccinated at an early age reach adulthood [9].

It is expected that unless this is done, the protection in adolescents will wane dramatically in the next few years due to different reasons. The seroprotection from the catch-up campaign conducted more than 10 years ago is waning, especially in subjects vaccinated at an early age.

If adolescents and young adults become unprotected, there is a risk of having a new peak in MenC incidence, not only in this age group but also in the rest of the Community, if the bacteria recirculate again. The development of an optimum strategy for the immediate future is a pressing public health need, and in this context the present study proposes an agent-based model for developing such a strategy and evaluating its impact.

## Methods

### Seroepidemiological study

Blood samples were collected from subjects over three years of age in different health centers of the province of Valencia (Spain), with an urban and rural distribution considered representative of the Community. Some samples were also collected from the largest hospitals in Valencia. All subjects or their parents/tutors signed an informed consent. The study was approved by the Ethics Committee of the Dirección General de Salud Pública/CSISP, Valencia. Samples were stored frozen at −80 °C and sent to the national meningococcal reference laboratory in Madrid, where SBA using rabbit complement was performed as described elsewhere [10].

Thirteen groups of subjects were studied, each representing a vaccination program at different ages: 3-4 years, 5-6 years, 7-8 years, 9-11 years, 12-13 years, 14-16 years, 17-19 years, 20-21 years, 22-29 years, 30-39 years, 40-49 years, 50-59 years, and over 60 years. No children under three years of age were studied, since the immunity of this age group could be estimated from the clinical trials and vaccine coverage.

The MenC vaccination status was reviewed for each participant in the Vaccine Information System (VIS) of Valencia [11].

### Estimation of waning immunity

Linking the seroprotection data to the patient vaccine status in the VIS, we were able to plot an evolutive seroprotection curve. We considered seroprotection when SBA ≥ 1:8. Subjects were vaccinated at different ages and with different schedules, all of which were taken into consideration. The vaccine trademark was very closely related to the age groups and schedules; consequently, no differences among the vaccines used in Valencia were taken into account.

### Agent-based model

We developed an agent-based model to simulate the evolution of the seroprotection histogram for the different age groups in the future, with the current vaccination schedule and with modified schedules. Our objective was to establish the best strategy based on two criteria: (i) seroprotection levels of infants and toddlers under two years of age are over 60*%*; and (ii) seroprotection levels of adolescents between 10-19 years of age are as high as possible.

**Figure 1:**
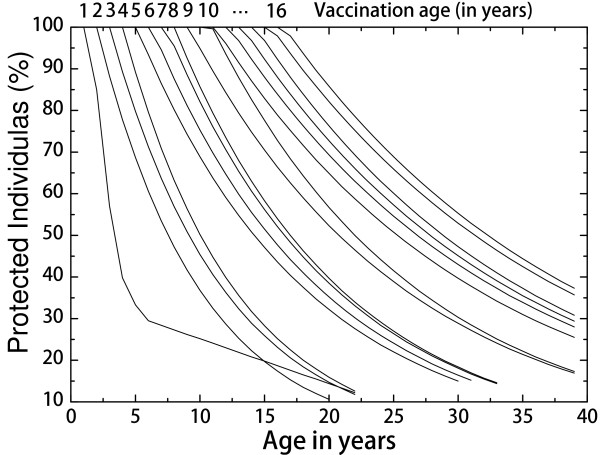
Plot displaying the fitted seroprotection levels in terms of age at the time of vaccination and age at the time of serological study.

We considered one million individuals represented by nodes and distributed according to the demographic data for the Community of Valencia [12]. The underlying demographic model was simulated in such a way that the total population remained constant: (i) individuals grew older according to the time-step used in the computer simulation (every step corresponding to one month in real time); (ii) a fraction of individuals died according to the mortality rate corresponding to their age; and (iii) deceased individuals were replaced by unprotected newborn infants.Seroprotection of the individuals (SBA ≥ 1:8) was a logical variable which evolved stochastically, i.e., after vaccination the individuals lose their immunity with a probability given by the seroprotection curve in Figure 1. Thus, we had to take into account the age at which the individual at a given node was vaccinated, because the waning of seroprotection depends upon the moment of vaccination. This variable was also stored by the computer program. Newborn infants were assumed to be unprotected.

Age groups were distributed in the same way as in the seroepidemiological study.The model proceeded as follows: To provide a stable demographic state, we assigned an age to each node according to the population histogram of the Community of Valencia. Taking into account the mortality rate per age group, we evolved this situation for a long enough time until the population histogram remained stable. Coherent mortality rates required that the final population state should not deviate significantly from the present state, as is in fact the case. Once the demographic model was established, we assigned the seroprotection label according to the study. A percentage of nodes were labeled as protected according to the fraction of protected individuals found in the seroprevalence study. When an individual was vaccinated, he or she became seroprotected (we did not consider vaccination failures). The initial state was known because we could classify the individuals according to their vaccination status (primovaccinated, booster or catch-up) and the time elapsed from the last vaccine dose. Starting with this initial condition, we took into account that with each time-step, each individual loses his or her immunity according to the decaying probability distributions shown in Figure 1, depending on the age at the time of the last vaccination. For each individual we also checked whether he or she was vaccinated in a scheduled vaccination campaign, and set the vaccination clock to zero in order to follow the evolution of seroprotection in the future.

An important point is the natural immunity of the unvaccinated population. A fraction of unvaccinated individuals was found to be protected, and this fraction increased with age. Consequently, in the model we considered this constant fraction (Table 1) to be protected and not to develop meningococcal C disease.

**Table 1:** **Fraction of unvaccinated individuals with *S B A *** **≥1:8 as a function of age group **[13]

**Age-group**	**% Seroprotection**	**CI 95%**
3-4 years	2.0%	[ 0.1*%*,5.8*%*]
5-6 years	4.0%	[ 0.7*%*,8.2*%*]
7-8 years	5.6%	[ 2.1*%*,9.8*%*]
9-11 years	6.6%	[ 2.9*%*,11.0*%*]
12-13 years	8.2%	[ 4.0*%*,12.6*%*]
14-16 years	9.6%	[ 5.2*%*,14.3*%*]
17-19 years	11.0%	[ 6.4*%*,15.6*%*]
20-21 years	12.3%	[ 7.8*%*,16.8*%*]
22-29 years	13.7%	[ 9.4*%*,18.0*%*]
30-39 years	15.2%	[ 11.4*%*,19.2*%*]
40-49 years	16.2%	[ 12.3*%*,20.5*%*]
50-59 years	18.1%	[ 13.5*%*,23.3*%*]
≥ 60 years	26.6%	[ 20.2*%*,34.1*%*]

The seroprotection of the vaccinated population never drops below the natural immunity levels in Table 1, assuming that the natural immunity is constant over time.

## Results and discussion

### Waning MenC seroprotection

The seroprotection study provided a snapshot of antibody persistence in the Valencian population in 2011 based on the VIS. We fitted an evolutive curve for seroprotection levels. Individuals with SBA ≥ 1:8 were considered protected against Neisseria meningitidis C disease. Since early studies, it has been known that SBA levels decrease very fast for children, but persist for longer times in adolescents [8]. The most recent study supports these early results and clearly shows that seroprotection wanes slower as the age of the vaccinated individual increases. A remarkable difference has also been observed between primovaccinated subjects (children under one year of age) and those individuals who received from 2006 a booster dose.

Figure 1 shows the results of the fitting procedure. The vertical axis represents the percentage of protected individuals, while the horizontal axis corresponds to the age at the time of the SBA assay. The vaccination age is also shown on the top horizontal axis. This fit was obtained by assuming that 100*%* (maximum) seroprotection is achieved at the time of vaccination and persists for the first year after vaccination (one year being our time discretization for this study). A large goodness of fit was obtained with an exponential decay fitting. Further details are given in the complementary material on the project webpage [13].

Vaccination seroprotection wanes very fast for children under one year of age, while among children from one year to 16 years of age the seroprotection period is considerably larger and increases steadily with age.

Thus, we should distinguish infants (in whom any dose should be regarded as primovaccination) and children older than one year (in whom any dose is a booster dose or catch-up). For example, in children who received a MenC vaccine dose at 6 months, seroprotection levels (initially above 90*%*) decreased to below 50*%* only 10 months later. In contrast, in children vaccinated at one year of age, seroprotection persisted above 50*%* during at least four years. The seroprotection half-life was 7 years when the age at vaccination was 8 years old [13].

### Outcome of the present 2-6-18 months MenC vaccination strategy

**Figure 2:**
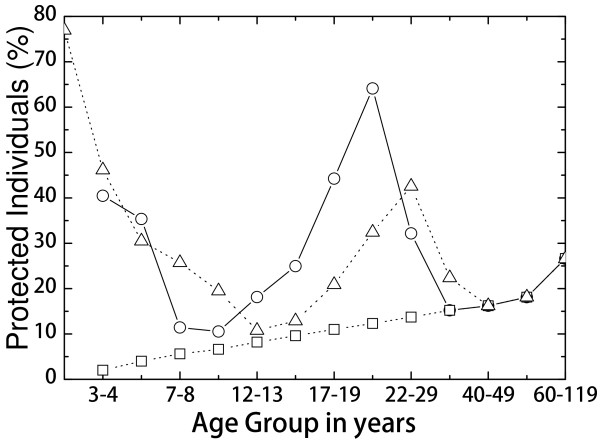
**Percentage of protected individuals in 2011 (circles) and October 2015 (triangles).** The squares correspond to the natural protection of unvaccinated individuals. The horizontal axis indicates the age group in years.

The seroprotection status in 2011 is shown in Figure 2. A broad seroprotection peak, over 50*%* in the those between 17 and 21 years of age, is a consequence of the catch-up program carried out from 2002 to 2004, when a large proportion of subjects between 6-19 years of age were vaccinated. Children under 3-4 years of age were also relatively well protected as a consequence of the booster dose in the second year of life. With the present vaccine schedule, at 2, 6 and 18 months of life, the predicted seroprotection level by age group in 2015 shows that the adolescents will become mostly unprotected, resembling the prevaccination situation. This is a consequence of increasing age and waning immunity.

### Strategy optimization

A public health goal is to keep adolescents and young adults well protected without lowering protection among those under four years of age. In order to optimize the seroprotection status, it might be desirable to change the vaccination schedule. To assess this issue, we considered that vaccines should be given on occasion of the pediatric monitoring visits recommended in the Community of Valencia (at 15 days, 1, 2, 4, 6 and 12 months of age, at 15 months and 18 months in the second year of life, and at 6, 12 and 14 years of age). Taking into account that such vaccination is not recommended below two months of age, we combined all the pediatric visits to define all the possible vaccination schedules with 1, 2 and 3 doses. This implied 129 different schedules. All of them were included in the model and simulated, starting in January 2014 and ending in November 2050. For each schedule we calculated the average protection for infants, toddlers (1-2 years of age) and teenagers between 10 and 19 years of age.

The four schedules that obtained the largest seroprotection level in each of the three time intervals are represented in Table 2. The three-dose schedule at 2 and 12 months of life and 12 years of age obtained the highest seroprotection level in the three age groups that were the objective of the highest seroprotection.

**Table 2:** Best vaccination schedules

**Order**	**Schedule**	**Avg. protect.**	**Avg. protect.**	**Avg. protect.**
		**0 y.o.**	**1 y.o.**	**10-19 y.o.**
1	2, 12, 144	63.84*%*	95.03*%*	62.62*%*
	months			
2	4, 12, 144	54.45*%*	95.03*%*	62.66*%*
	months			
3	2, 15, 168	63.80*%*	82.43*%*	55.18*%*
	months			
4	2, 15, 144	63.81*%*	82.41*%*	62.65*%*
	months			

The first column is the order. The second is the vaccination schedule. The third is the average protection of children less than a year old during the period January, 2014 (new vaccination schedule starts) until November, 2050. The fourth and the fifth show the same for one year old children and 10-19 years old adolescents, respectively.

**Figure 3:**
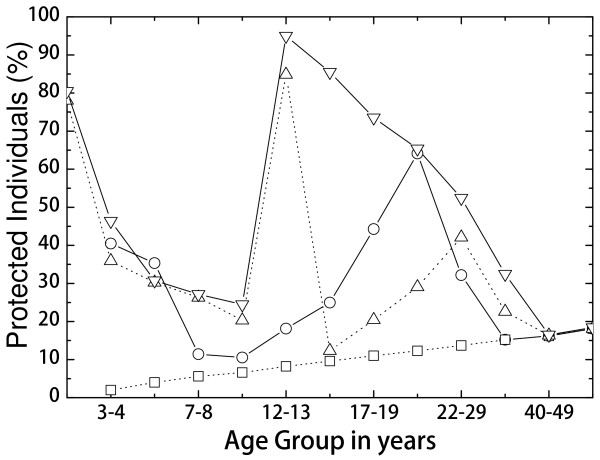
**Percentage of protected individuals with the 2-12-144 months strategy.** The circles are the result of the seroprevalence study in 2011, squares correspond to natural immunity, upward triangles reflect seroprotection in January 2016 (two years after implementation of the new strategy), and the downward triangles correspond to the long-term protection achieved in September 2040.

In relation to a change in vaccination schedule with a single priming dose in infants under one year of age, it should be noted that a very recent study clearly shows a single-dose priming vaccination to be a valuable and reasonable alternative to the current two-dose vaccination schedule [14].Figure 3 depicts the evolution of the seroprotection histogram with the best strategy. In the long term, this strategy achieves the highest protection levels for every age group.

However, before achieving this result, a transient situation is observed in which adolescents are poorly protected. In particular, we can see that in January 2016 (only two years after starting the new vaccination schedule), the 14-16 year-old subjects are practically at levels of natural immunity. The reason for this behaviour is that boosting 12 year-olds have still not reached the 14-16 years of age group, while adolescents show a waning of their SBA levels.

### Complementary catch-up

To overcome this situation, even with a new booster dose at 12 years of age, there might be a need to perform a catch-up program together with the booster. The target population for the catch-up at least should comprise subjects aged 13-15 years in January 2014.

**Figure 4:**
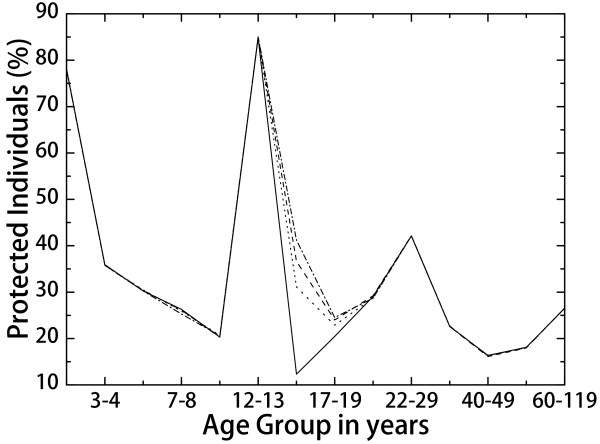
**Percentage of protected individuals with the 2-12-144 months strategy and a catch-up program targeting the 13-15 year-old subjects from January 2014 to December 2014.** The solid line is the protection histogram in January 2016 without catch-up, the dotted line correspond to the result with a catch-up targeting 30*%* of the population in that group, the dashed line stands for 40*%*, and the dashed-dotted line stands for 50*%*.

In order to maintain good indirect protection, the objective of the catch-up could not be reaching the whole population. We therefore considered three different catch-up scenarios, of different intensity, targeting these age groups. The results are shown in Figure 4. Even with a 30*%* catch-up coverage, a very significant improvement in protection of the 14-16 year-old group is obtained. In this case the protection by January 2016 (just two years after starting the new schedule) is around 40*%*, compared with 10*%* in the absence of any catch-up campaign. In the long run, the protection histogram will be the same as that in Figure 2 for September 2040.

## Conclusions

In this study we considered the scenario following a vaccination schedule change in Spain in the near future. A snapshot of the seroprotection levels for different age groups in the Autonomous Community of Valencia (Spain) was obtained in a seroepidemiological study carried out in 2011. By crossing these data with the vaccination records, this detailed study allowed us to fit an accurate waning seroprotection table for conjugate meningococcal C vaccination. This study is not only important for public health decisions in the immediate future in Spain [15] but also for other countries which have shifted from polysaccharide to conjugate vaccination in the last decade [16-18]. Nowadays, the situation referred to seroprotection in the Autonomous Community of Valencia can be considered very good, as reflected by the rareness of cases of meningococcal C disease. The explanation for this effective control can be found in the high serum bactericidal antibody levels in both adolescents and children under four years of age. High SBA levels in adolescents from Valencia in 2011 are the direct consequence of a high coverage catch-up campaign carried out in 2000-2004, in which subjects from one year to 20 years of age were targeted.

Although MenC disease is now rare in Valencia, the waning of SBA levels in the near future, especially in the adolescent population, may imply the recirculation of MenC. We have quantitatively shown that the 14-16 years age group will become at high risk of contracting meningococcal disease in 2016, compared with the pre-vaccination era incidence. This situation has motivated a plan for changing the vaccination schedule from the three doses administered at 2, 6 and 18 months of age to another schedule in which one of these doses is removed and displaced to late childhood (some moment between 11 and 14 years of age). This optimization problem can be discussed in the framework of an agent-based model, and the best strategy can be defined and selected according to the levels of protection for adolescents and children ranging from newborn infants to two-year-old toddlers. We sought a strategy protecting the adolescents from 10 to 19 years of age with a minimum average of 50*%*, while at the same time keeping infants with seroprotection levels as high as possible.

Checking all possible combinations for three doses, we found the optimum strategy to correspond to vaccination at 2 months, 12 months and 12 years of age. With this strategy, infants are protected (with 50*%* probability) for at least 5 years. A second booster dose for 12 year-olds protects them during the adolescence period. Moreover, this booster dose could be planned easily because, at that age, the penultimate pediatric monitoring visit is scheduled in Spain. The simulation shows that the protection levels after several decades will rise to levels above the 2011 histogram for all the target groups.

However, that change in schedule will not be fully effective in the first years, since adolescents from 15 to 19 years of age keep waning their seroprotection, and will not be included in the booster campaign. With only a 20*%* seroprotection level in this age group in 2016-2019, recirculation of MenC in the community could occur. The model therefore anticipates that the change in vaccination schedule should be accompanied by a catch-up campaign in adolescents from 13 to 15 years of age.

It should be mentioned that the model has some limitations. We considered that a booster dose in adolescents would protect these subjects for the same time period as primovaccination given at the same age. However, the serological response could be different, though only an unexpected short duration of immunity would affect the model. We also considered that catch-up would be given in a random fashion. However, it is possible that unvaccinated collectivities might appear, with an unknown effect upon the recirculation of MenC.

The percentage of the population that must be protected in order to prevent MenC circulation is not clear. Trotter et al. [19] postulated that protection would occur with an immunization threshold in the range of 17−26*%*; this is the reason why we consider than even a low coverage catch-up campaign would be efficacious. The model allowed us to estimate the minimal vaccination coverage in the catch-up campaign.

## Competing interests

Dr. Javier Díez-Domingo has been a Principal Investigator in clinical trials for Baxter and Novartis. He has also given conferences sponsored by Baxter and Novartis.

## Authors’ contributions

LPB participated in the seroepidemiological study and data retrieval. RJV and JM contributed to the agent-based mathematical model and the data analysis. JVO developed the distributed computing system for model simulations. LA participated in the design and analysis of the mathematical model and wrote the paper. FS participated in the statistical data analysis and model development. RA participated in sample collection and laboratory analysis. JAV and JDD coordinated the project and participated in every stage of the work. All the authors have read the manuscript and approve it.

## Pre-publication history

The pre-publication history for this paper can be accessed here:

http://www.biomedcentral.com/1471-2334/14/280/prepub
